# Human fMRI Reveals That Delayed Action Re-Recruits Visual Perception

**DOI:** 10.1371/journal.pone.0073629

**Published:** 2013-09-06

**Authors:** Anthony Singhal, Simona Monaco, Liam D. Kaufman, Jody C. Culham

**Affiliations:** 1 Department of Psychology and Centre for Neuroscience, University of Alberta, Edmonton, Alberta, Canada; 2 Centre for Vision Research, York University, Toronto, Ontario, Canada; 3 Brain and Mind Institute, Department of Psychology, University of Western Ontario, London, Ontario, Canada; National Institute of Mental Health, United States of America

## Abstract

Behavioral and neuropsychological research suggests that delayed actions rely on different neural substrates than immediate actions; however, the specific brain areas implicated in the two types of actions remain unknown. We used functional magnetic resonance imaging (fMRI) to measure human brain activation during delayed grasping and reaching. Specifically, we examined activation during visual stimulation and action execution separated by a 18-s delay interval in which subjects had to remember an intended action toward the remembered object. The long delay interval enabled us to unambiguously distinguish visual, memory-related, and action responses. Most strikingly, we observed reactivation of the lateral occipital complex (LOC), a ventral-stream area implicated in visual object recognition, and early visual cortex (EVC) at the time of action. Importantly this reactivation was observed even though participants remained in complete darkness with no visual stimulation at the time of the action. Moreover, within EVC, higher activation was observed for grasping than reaching during both vision and action execution. Areas in the dorsal visual stream were activated during action execution as expected and, for some, also during vision. Several areas, including the anterior intraparietal sulcus (aIPS), dorsal premotor cortex (PMd), primary motor cortex (M1) and the supplementary motor area (SMA), showed sustained activation during the delay phase. We propose that during delayed actions, dorsal-stream areas plan and maintain coarse action goals; however, at the time of execution, motor programming requires re-recruitment of detailed visual information about the object through reactivation of (1) ventral-stream areas involved in object perception and (2) early visual areas that contain richly detailed visual representations, particularly for grasping.

## Introduction

Grasping an object no longer in view requires the motor system to access stored information about the size, shape, position, and orientation of the intended object to guide and preshape the hand. Memory-guided grasping is distinct from immediate grasping, where accurate information about relevant object properties is continuously provided by the visual system to the motor system on a moment-to-moment basis.

Considerable research has investigated the neural substrates of immediate actions. In an influential model of perception and action [1], the control of action is thought to be mediated by the dorsal stream in occipital-parietal cortex while perceptual object processing is thought to be mediated by the ventral stream in occipital-temporal cortex. For example, visually guided grasping recruits the anterior intraparietal sulcus, aIPS [2], [3]; whereas, visual recognition recruits the lateral occipital cortex, LOC [4], [5].

Far less is known about the neural substrates of memory-guided actions, although neuropsychology and behavioral studies have suggested they may rely on somewhat different mechanisms than immediate actions. Neuropsychology has revealed a double dissociation between the ability to perform immediate vs. delayed actions. Specifically, a patient, DF, with visual form agnosia and bilateral lesions within LOC [6] was far more impaired in performing delayed than immediate actions [7], [8]. Conversely, another patient, IG, with optic ataxia and a lesion to posterior parietal cortex, performed better for delayed than immediate actions [9]. Other dissociations between delayed and immediate actions have been observed in healthy normal participants [10]. For example, a number of studies have reported that grasping is more susceptible to illusions when relying on remembered vs. online visual information [7], [11], [12].

Taken together, this evidence has been used to argue that memory-driven actions rely more on the ventral visual stream than immediate actions because of different computational requirements [10]. Specifically, the dorsal-stream processing of actions in real-time is thought to rely on in-the-moment computations of the absolute metric properties in egocentric coordinates, whereas the ventral stream processing in delayed actions is thought to utilize relational metric properties in scene-based coordinates.

However, not all research supports this model. Some behavioral findings have been used to argue against a strict dichotomy [13]–[15] or an abrupt transition between mechanisms [16]. Moreover, the accuracy of delayed actions is generally worse for patients with optic ataxia than healthy control subjects [17] and can be disrupted by parietal stimulation [18]. In addition, single neurons in the macaque anterior intraparietal area show sustained activity during delays [19] and human neuroimaging suggests similar effects in aIPS [20]. Indeed fMRI studies to date have found strikingly similar activation when comparing real and delayed actions, including motor cortex, premotor regions and posterior parietal regions as well as cerebellum [20], [21].

We used neuroimaging in healthy normal participants to investigate the contributions of the dorsal and ventral streams during initial object presentation, an 18-s delay, and execution of grasping or reaching actions. This long-delay design enabled us to clearly distinguish activation related to visual stimulation/encoding, memory maintenance during the delay period, and memory retrieval at the time of execution, thus providing a richer characterization of dorsal and ventral stream brain regions to delayed actions.

## Materials and Methods

### Participants

**Figure 1:**
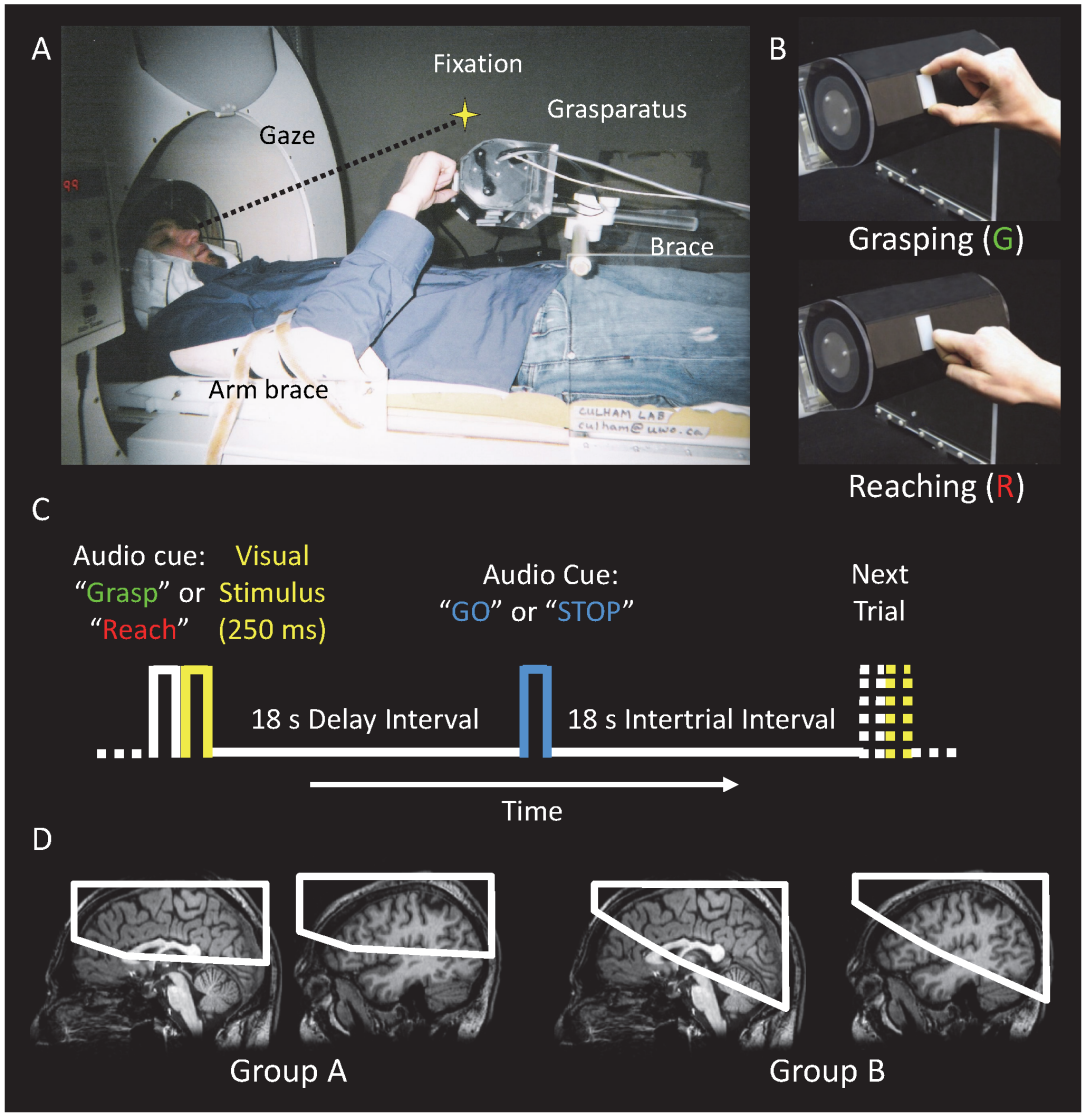
Experimental Paradigm. (**A**) The participant’s head was tilted to enable fixation upon an LED placed on top of the magnet bore, such that the stimuli were viewed in the lower visual field. The right upper arm was restrained in an arm brace to minimize shoulder movements. The participant shown provided written informed consent, as outlined in the PLOS consent form, to publication of his photograph. (**B**) In grasping trials, participants performed a precision grip with the finger and thumb along the long axis of the object. In reaching trials, they simply touched the object with the knuckles. Although the hand and object are simultaneously visible in the image, this was not the case in the actual experiment. (**C**) Each trial began with an auditory cue that instructed the participant about the task on the upcoming trial (“Grasp” or “Reach”) after which the object was illuminated for 250 ms. Following an 18- s delay, an auditory cue instructed the participant to either execute the movement (“Go”) or abort any response (“Stop”). Following the brief visual stimulus, the participant remained in complete darkness during the delay and action periods. (**D**) Functional slice planes differed between two groups of participants. Group A (left) consisted of 11 participants scanned with a slice orientation that covered most of the frontal lobe and the entire parietal lobe. Group B (right) consisted of 9 participants scanned with a slice orientation that covered the parietal and occipital lobes and part of the temporal lobes. For each group, the white box indicates the functional volume common across all subjects within the group (defined in Talairach space) superimposed on midsagittal and parasagittal (through LOC) slices of a representative participant’s anatomical scan.

Due to the limited number of slices that could be collected in a 2-s volume acquisition time on the high-field MRI scanner we employed, data from two groups were collected with two different slice orientations, one to collect frontal and parietal and data (n = 11) and one to collect also occipital and temporal cortex data (n = 9) (Figure 1D). Eight participants overlapped between Group A and Group B. All participants were right-handed and had normal or corrected-to-normal vision. The experiment was approved by the Health Sciences Research Ethics Board (Protocol #13507) at the University of Western Ontario based on principles consistent with the Declaration of Helsinki. Participants provided written consent and completed an MRI safety screening form prior to testing.

### Procedure

**Figure 2:**
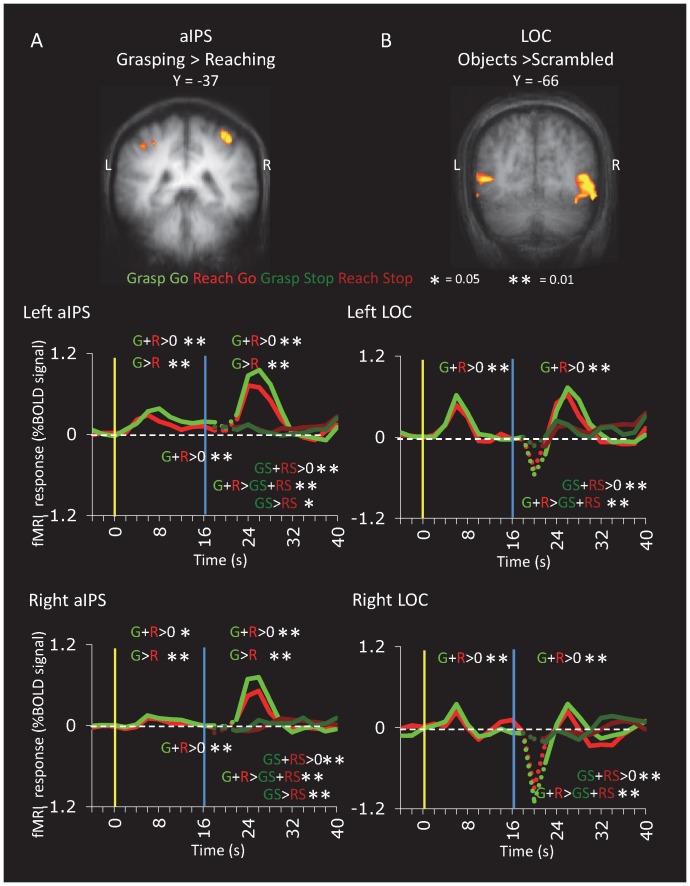
Voxelwise Maps and Region of Interest Time Courses for aIPS and LOC. Activation maps show group activation in aIPS, identified by Grasping>Reaching in Group A and in LOC, identified by Objects>Scrambled in Group B (RFX GLM contrasts; t >3 for aIPS; t >4.5 for LOC, corrected). Activation is superimposed on the group-averaged anatomical slices. Although group data is presented to illustrate the average location of the areas, time course data and statistical comparisons between conditions were performed on regions of interest extracted from individual subjects based on data from independent localizer runs. In individuals, areas aIPS (**A**) could be identified in the 9 of the 11 participants of Group A and LOC (**B**) could be identified in all 9 participants of Group B. In the time courses, the yellow vertical line indicates the time of the visual stimulus and the blue vertical line indicates the time of the action cue. Note that Go and Stop trials were indeterminate until the action cue; thus, time course graphs show only grasp (green) and reach (red) lines prior to the action cue (blue line). The Grasp Go (light green) vs. Grasp Stop (dark green) and Reach Go (light red) vs. Reach Stop (dark red) curves diverge after the action cue (blue line). Time points for which artifacts occurred for the Grasp Go and Reach Go events are indicated by dotted lines. For each area in each hemisphere and for each phase (Vision, Delay, Go, Stop), we tested whether the beta weights were significantly higher than baseline activation, whether activation was higher for grasping than reaching, and whether Go activation was higher than Stop activation. Only significant effects are reported.

We used high-field (4-Tesla) functional magnetic resonance imaging (fMRI) to measure the blood-oxygenation-level dependent (BOLD) signal [22] in a slow event-related delayed action paradigm. As shown in Figure 1A, participants performed delayed hand actions upon three-dimensional (3D) target stimuli located in the workspace of the hand below a fixation point that was viewed directly without mirrors [3]. Each trial sequence (Figure 1C) consisted of: (1) an auditory cue to “Grasp” (perform a reach-to-grasp movement with a precision grip with the finger and thumb along the long axis of the object, requiring both arm transport and hand preshaping) or “Reach” (reach-to-touch movement to contact the object with the knuckles, requiring arm transport without hand preshaping) on the upcoming trial (Figure 1B); (2) a brief (250-ms) illumination of the target object; (3) a long delay interval of 18 s; (4) an auditory cue to perform the cued action upon the object based on the information held in memory (“Go”) or to simply abort the action (“Stop”); and (5) an intertrial interval (ITI) of 18 s before the next event. Activation following the Go cue could have been related to action execution, memory recall, or simply the end of the trial [23]. To distinguish the first two possibilities from the last, we included the Stop trials in which the trial was aborted, but with no action performed and thus no need to recall the stimulus.

Although behavioral research has shown that brief delays of 2 s [7] or even a fraction of a second [24] can change the nature of processing, pilot testing of fMRI data with shorter (10-s) intervals [25] revealed that it was very difficult to dissociate delay period activation from the preceding visual stimulation period and the subsequent action period. In such cases, erroneous delay activation can arise due to imperfections in the hemodynamic model, particularly given its variability between subjects [26]. As such, we utilized a relatively long delay interval of 18 s to isolate delay-period activation unequivocally.

Importantly, aside from the brief period of visual illumination, participants remained in complete darkness, except for a small light emitting diode (LED), which was too dim to allow vision of anything else within the scanner bore. Reaction time (RT) and accuracy for Go responses were collected when the participant released a key placed on the torso.

In sum, the 2×2 factorial design, with factors of task cue (Reach or Grasp) and execution cue (Go or Stop) led to four trial types: Grasp Go, Grasp Stop, Reach Go and Reach Stop. Because Go and Stop periods were only distinguished at the end of the delay period, prior to that the data were grouped into just two categories, Grasp and Reach.

There were 16 trials per experimental run, with each of the four trial types presented in counterbalanced order for a run time of ∼11 min. Participants completed on average 106 trials (min 64 trials, max 160 trials).

### Apparatus and Stimuli

3D objects were presented on the original “grasparatus” grasping apparatus developed within our lab [3]. The grasparatus is a non-metallic octagonal rotating drum that is pneumatically driven to present 3D target stimuli on each of the eight faces (Figure 1A and 1B). In this experiment, the targets were rectangular translucent plastic objects that varied in size, spatial position, and orientation. Objects had a width of 16 mm and a depth of 6 mm but varied in length (16 to 40 mm in 3-mm steps for a total of 8 lengths). The objects could be placed at one of four locations, 34 or 84 mm to the left or right of the grasparatus centre which was positioned below the fixation light. Objects could also vary in orientation, although only within certain ranges to permit a comfortable grip. For each participant, the experimenter placed the objects on the grasparatus in a random orientation and then cycled through the faces, giving the participant the opportunity to adjust any orientations that felt awkward. The targets were illuminated by light-emitting diodes (LEDs) mounted beneath the each object. Participants grasped the object along the long axis using a precision grip with the finger and thumb but could not lift the objects off the surface of the grasparatus. Participants were instructed to grasp the object (without making corrections if the grasp was imperfect) and then return to the home position.

The participants lay in the magnet with their heads and torsos tilted (∼30 deg.) to allow direct viewing of the target area without the use of mirrors. The right upper arm was restrained with an arm brace to restrict shoulder movements, but allowed for full motion of the elbow and wrist. The grasparatus was placed above the participant’s hips to allow comfortable reaching and grasping from a starting position on the chest where the right index finger was placed over a key-press.

We are not able to use an MR-compatible eye tracker with the head-tilted configuration because the eyelids droop in that posture, occluding too much of the pupil to detect eye position; nevertheless, our subjects were highly experienced fMRI participants who were accustomed to the requirement to maintain fixation throughout fMRI runs. Moreover, eye tracking outside the scanner showed that a similar group of subjects could successfully fixate throughout a run with negligible eye movements and no differences between grasping and reaching conditions [27].

### Localizer Scans

To localize visuomotor areas, we included runs of immediate grasping and reaching without visual feedback (open loop) in 9 of 11 participants of Group A (for 2 of 11 participants, insufficient time was available to include these localizer scans). To localize the object-selective lateral occipital cortex, we obtained data comparing objects vs. scrambled objects [28] that was aligned to the experimental session.

### Imaging Parameters

Scanning was done in a 4-Tesla whole-body MRI system (Siemens-Varian) at the Robarts Research Institute (London, ON, Canada) employing a single-channel transmission-reception cylindrical birdcage radiofrequency whole-head coil. Functional volumes were collected using a T2*-weighted, segmented gradient-echo echoplanar imaging (19.2 cm field of view with 64×64 matrix size for an in-plane resolution of 3 mm; repetition time, TR = 1 s, with two segments/plane for a volume acquisition time of 2 s; time to echo, TE = 15 ms; flip angle, FA = 45 deg.). Each volume was made up of 15 contiguous slices of 5-mm thickness. For Group A, slices were angled at approximately 15 deg. from axial to cover entire parietal cortex and superior frontal cortex, and for Group B, slices were angled at approximately 30 deg. from axial to cover the entire parietal cortex, entire occipital cortex, posterior temporal cortex and posterior frontal cortex (Fig. 1D). High-resolution T1-weighted anatomical volumes were acquired along the same orientation as the functional images using a 3D acquisition sequence (256×256×64 matrix, 0.75 mm in-plane resolution, 3 mm slice thickness, T1 = 600 ms, TR = 11.5 ms, TE = 5.2 ms, FA = 11 deg.).

### Data Processing and Analysis

The imaging data were preprocessed and analyzed using Brain Voyager software (BV QX 1.10, Brain Innovation, Maastricht, The Netherlands). Anatomical volumes were transformed into standard stereotaxic space [28]. Functional data were preprocessed with linear trend removal followed by temporal high-pass filtering set to remove frequencies below 2 cycles/run. For each participant, functional data from each session were screened for motion or magnet artifacts with cine-loop animation. There were no abrupt movements exceeding 1 mm or 1 deg. and no obvious artifactual activation (such as rims of false activation) in the statistical maps for the contrasts performed for any subject. Because head motion was negligible and motion correction can lead to artifactual activation [29], no motion correction was applied. Data was spatially smoothed (full-width, half-maximum, FWHM = 4 mm) for group but not individual participant analyses.

Data were analyzed using a random effects (RFX) general linear model (GLM) with separate predictors for each phase of each trial type: Reach-Visual, Reach–Delay, Reach–Go, Reach-Stop, Grasp-Visual, Grasp-Delay, Grasp-Go, Grasp-Stop. The Visual, Go, and Stop predictors were modeled with a single-volume (2-s) rectangular wave, and the Delay predictors were modeled with a 9-volume (18-s) rectangular wave that was sustained throughout the delay period. Each predictor was convolved with a standard hemodynamic response function (HRF; Boynton model). Although the visual stimulus lasted less than 2 s (250 ms), we found that the predicted HRF for a 2-s Visual predictor demonstrated a good fit with visual response seen in the event-related average time courses across brain areas, perhaps because sensorimotor processing continued beyond visual stimulation (e.g., iconic memory, visual encoding, motor planning). Similarly, the predicted HRF for the Go predictor demonstrated a good fit with the execution response seen in the event-related average time courses across brain areas. Time course data were z-transformed prior to analysis. Since conditions were distributed equally within runs (and thus had the same standard deviation), the beta weights reflect the magnitude of activation.

During the action phase in the Go conditions, the hand actions sometimes caused a distortion of the magnetic field observed as negative spikes during the first volume of each event. These motion artifacts occurred abruptly and without the standard hemodynamic lag (∼ 5 s) and response profile. To adequately account for the variance due to these artifacts, the GLM included two single-volume spike predictors of no interest (one for Reach-Go and one for Grasp-Go) that peaked one volume after the go cue (corresponding with the spikes that can be observed as for example in the LOC time courses in Figure 2).

To correct for the problem of multiple comparisons, we used Brain Voyager’s cluster-level statistical threshold estimator plug-in. This algorithm uses Monte Carlo simulations (1000 iterations) to estimate the probability of clusters of a given size arising purely from chance. Because the minimum cluster size for a corrected p value is estimated separately for each map (based on smoothness estimates), cluster sizes can vary across different comparisons. Nevertheless all the clusters reported have a minimum size of at least 6× (3 mm)^3^ = 162 mm^3^. All post hoc contrasts between conditions were performed using Brain Voyager’s region-of-interest general linear model (ROI-GLM) feature with RFX to compute statistical significance based on beta weights within each region.

## Results

We used RFX-GLM contrasts to (a) identify, at the group level, key regions for which we had hypotheses (including those derived from independent localizers); and (b) perform an exploratory search of activations for key experimental contrasts. To show the patterns of data across the phases and tasks, we have plotted event-related average time courses and performed statistical comparisons between critical conditions in each phase.

To avoid issues with analyses that are non-independent with respect to the selection criteria, we have used several approaches. Where possible, we have used selection criteria independent from the key experimental contrasts. Where that was not possible, we have used square brackets to flag contrasts that are non-independent of the means used to select the regions so the reader is aware of those cases. Averaged Talairach coordinates are provided in Table 1.

**Table 1:** Talaraich coordinates of brain areas activated in eight contrasts.

	Talairach Coordinates		
	X	Y	Z
A. Localizer: Immediate Grasping>Reaching (Group A, n = 9/11, t>3)			
Left aIPS	−39	−34	45
Right aIPS	37	−37	52
B. Localizer: Objects>Scrambled Objects (Group B, n = 9, t>4.5)			
Left LOC	−47	−66	1
Right LOC	46	−65	2
C. Conjunction of Action Reactivation and Object Selectivity (Group B, n = 9, t >3 per contrast)			
Left Overlap Region	−51	−58	1
Right Overlap Region	50	−55	4
D. Experimental Runs: (Grasp Vision+Reach Vision)>Baseline (Group B, n = 9, t>5.5)			
Left cuneus	−5	−79	14
Right cuneus	3	−82	7
E. Localizer: Immediate Actions>Baseline (Group A, n = 9/11, t>8.5)			
Left SPOC	−4	−84	30
F. Experimental Runs: (Grasp Vision+Reach Vision)>Baseline (Group A, n = 11, t>5)			
Pre-SMA	0	11	53
Left PMd	−28	−13	55
Right PMd	23	−9	49
Left IPS	−38	−49	46
	−27	−55	45
Right IPS	25	−51	45
Right precuneus	12	−73	43
	20	−70	36
	3	−70	43
Midline cuneus	−2	−74	14
Left LOC/MTG	−47	−62	2
Right LOC/MTG	41	−66	8
G. Experimental Runs: Action Reactivation>Stop Reactivation (Group B, n = 9, t>5)			
Pre-SMA/SMA	−5	−3	47
Left PMd	−25	−18	63
Right PMd	24	−13	61
Left mid-precentral sulcus	−45	−1	40
Left insula	−37	2	13
Left M1	−44	−21	55
Left IPS	−47	−35	55
	−32	−49	53
Right IPS	30	−51	56
Left SII	−60	−24	26
Right SII	57	−19	28
Left lateral SPOC	−14	−79	36
Right lateral SPOC	9	−75	35
Right precuneus	6	−72	45
	4	−60	52
	3	−50	58
Left posterior cingulate sulcus	−11	−32	43
Right posterior cingulate sulcus	9	−32	52
Left LOC/MTG	−53	−62	−1
Right LOC/MTG	51	−55	4
Anterior calcarine sulcus	0	−66	9
Posterior calcarine sulcus	0	−80	1
Left thalamus	−13	−20	14
Right thalamus	12	−16	14
Superior cerebellum	−2	−55	−8
H. Experimental Runs: (Grasp Delay+Reach Delay) vs. Baseline (Group A, n = 11, t>3.2)			
Left M1	−39	−19	51
	−34	−33	53
SMA	−3	−15	55

The contrasts used to define these areas are indicated with letters from A to H. Area abbreviations: aIPS, anterior intraparietal sulcus; LOC, lateral occipital complex; SPOC, superior parietal occipital sulcus; SMA, supplementary motor area; PMd, dorsal premotor; M1, primary motor; IPS, intraparietal suclus; SII, secondary somatosensory; MTG, middle temporal gyrus.

### Behavioural Data

The behavioural data confirmed that the participants were executing the task correctly with respect to the “Go vs. Stop” conditions. The RT data revealed no significant differences between grasping and reaching conditions (grasping RT = 321.6 ms +/−9.8 (SE); reaching RT = 322.7 ms +/−10.6 (SE); t(10) = 0.896, p = 0.39 two-tailed).

### Region-of-Interest Analyses

#### Area aIPS

Area aIPS was localized at the junction of the intraparietal and postcentral sulci using an independent contrast of Immediate Grasping>Immediate Reaching (t>3) from separate runs in 9 out of 11 participants of Group A. Time courses and post hoc analyses showed that in both hemispheres, aIPS showed significant activation relative to the intertrial interval for all three phases of the sequence (Figure 2A, Table 1A). Moreover, activation was significantly higher for grasping than reaching in both the vision and action phases but not the delay phase. Activation for Go trials was considerably and significantly stronger than that for Stop trials.

These results demonstrate that human aIPS, like its putative macaque homologue, AIP, is sensitive to visual presentation (without simultaneous action), visuomotor delays, and reach and grasp actions in the dark (without simultaneous target vision or visual feedback). Moreover, the visual responses and grasp-selectivity during vision suggest that human aIPS is not merely activated by the greater somatosensory stimulation and motor demands of grasping vs. reaching (as for example is S1). Our results also strengthen the case for homologies between human aIPS and macaque AIP ([30]–[32] but see [33]) which also shows visual and motor responses [34] as well as sustained activation over a delay period [19].

#### Lateral Occipital Complex

Area LOC was identified inferior to the junction of the inferior temporal sulcus and lateral occipital sulcus using an independent contrast of Intact Objects>Scrambled Objects (t>4.5) in all 9 participants of Group B. Although activation of LOC during visual stimulus presentation was expected, we were surprised to observe a robust reactivation of LOC at the time of action (larger for Go than Stop trials), even though participants were in complete darkness (Figure 2B, Table 1B). This “action reactivation” occurred despite the absence of a sustained signal during the delay period and was not significantly modulated by whether participants were performing a grasping or reaching action.

### Group Voxelwise Analyses of Regions-of-Interest

In addition to the ROI analyses above, we also conducted group voxelwise contrasts to further examine activation across these areas and others.

#### Lateral Occipital Cortex

**Figure 3:**
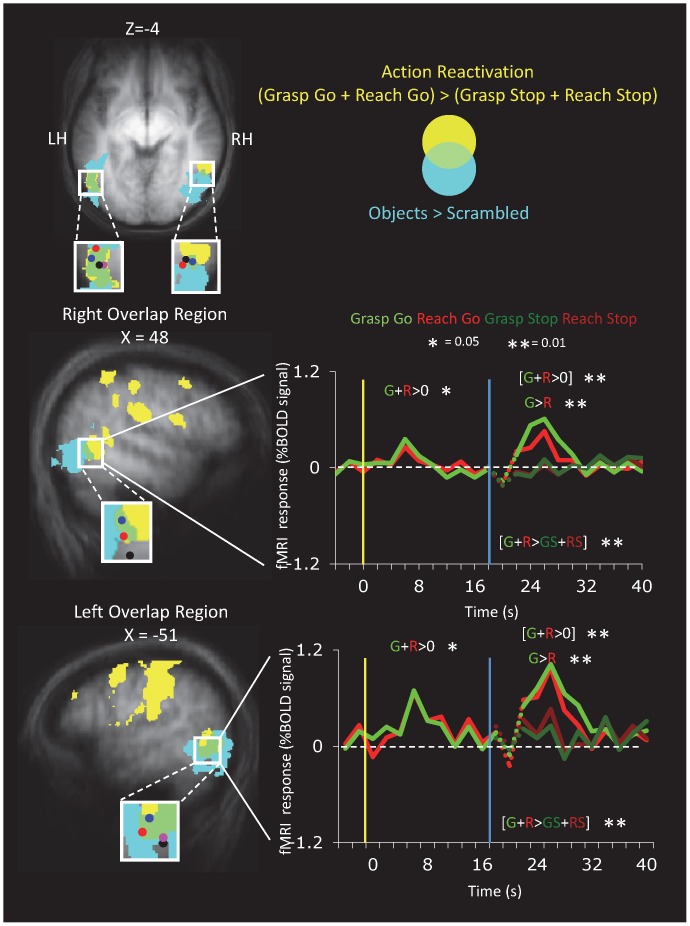
Voxelwise Temporal Lobe Activation in the Vicinity of the Lateral Occipital Complex. Activation maps show group activation from 9 participants of Group B on the group-averaged anatomical for different contrasts. Maps show significant effects for action reactivation (t >3, k = 10 voxels) in yellow and for the Object>Scrambled contrast (t >3, k = 9 voxels),in blue. The Venn diagram shows effects of overlapping activation colors. The time course is shown from the region of overlap of all three contrasts. Contrasts listed in square parentheses are expected given the criteria used to select the region. The blue dot indicates the Talairach coordinates of the overlapping region in the present study. Other dots indicate the Talairach coordinates of LO_TV_ from previous studies: Pink = Amedi et al. 2001; Black = Amedi et al. 2002; Red = Tal & Amedi 2009).

Although the ROI approach is valuable in evaluating responses in well-known areas, it neglects the possibility that in large areas such as LOC, only a subregion of the ROI may be activated in the experimental contrast. To investigate the pattern of activation within and around the LOC, we performed a voxelwise group analysis of activation overlap. As shown in Figure 3 (and Table 1C), we superimposed activation maps for object-selectivity in the LOC localizer (Intact Objects>Scrambled Objects, Group B) and the action reactivation in the experimental task (Grasp Go and Reach Go>Grasp Stop and Reach Stop, Group B). In the right temporal lobe, action reactivation was observed at the anterior end of the lateral LOC, rather than throughout the entire complex, extending anteriorly into the middle temporal gyrus (MTG). The area of overlap from these two contrasts has the properties expected of an area at the interface between action and perception; thus we took a closer look at its pattern of activation. In both hemispheres, not only did the region show action reactivation as expected by the selection criteria, but it also showed visual responses, and grasp-selectivity during the reactivation (though no significant delay period activity was evident). The location of this overlapping activity had similar Talairach coordinates as those reported for LO_TV_ [35]–[37], an area implicated in cross-modal tactile-visual integration (hence the TV subscript in its name).

#### Early Visual Cortex

**Figure 4:**
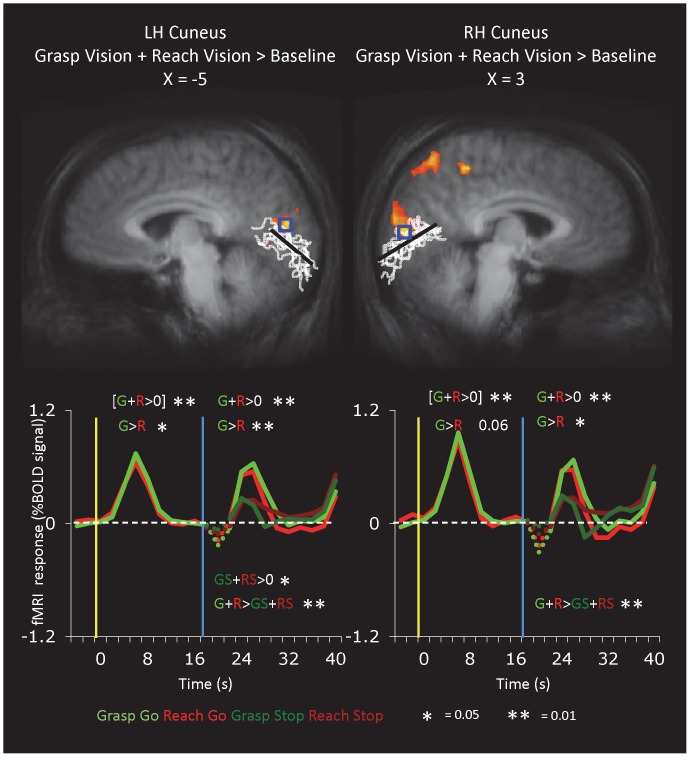
Re-Activation in Early Visual Areas at the Time of Action. The statistical maps show areas activated during the visual phase of delayed grasping in 9 participants of Group B (t>5, corrected). The white lines represent the calcarine sulcus from each of the nine participants while the black straight line approximates the calcarine sulcus on the group-averaged anatomical. Although retinotopic mapping data were not available, we selected a visually activated region in each hemisphere (blue box) that was near and slightly above the calcarine sulcus, where the representation of the lower visual field in area V1 would be expected, and extracted the time courses for further analysis.

Given the action reactivation observed in the vicinity of LOC, we wondered whether even early visual cortex (EVC) might reflect the same phenomenon. Although we did not have retinotopic mapping data for our participants, we identified the region of occipital cortex near the calcarine sulcus that showed the highest response for visual stimulus presentation [(Grasp and Reach)>Baseline, Group B], (Figure 4 and Table 1D). This focus was in the anterior, dorsal occipital cortex (cuneus) just above the average calcarine sulcus, consistent with the presentation of the visual stimulus in the visual periphery of the lower visual field. Talairach coordinates suggested that these regions fell within Brodmann area 17 and/or area 18 (V1 and/or V2 [38]). After identifying EVC in each of the two hemispheres based on its visual response, we then examined its time course. We found higher activation for grasping than reaching in both the visual phase (though in the right hemisphere, this was only a trend) and action phases, but no activation nor any grasp-selective activation in the delay or no-go phases.

#### Superior Parieto-occipital Cortex

**Figure 5:**
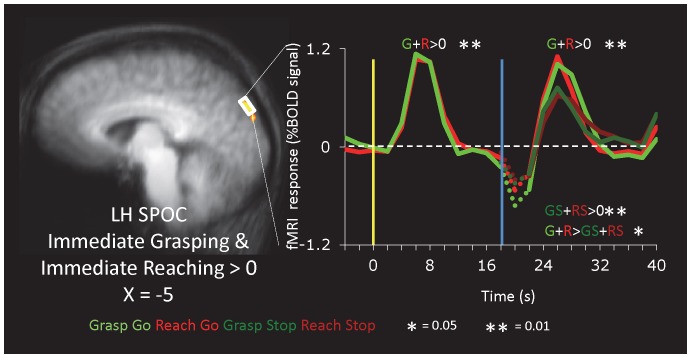
Activation in Superior Parieto-Occipital Cortex. SPOC was identified in Group A (n = 9) using a contrast of Immediate Grasping+Immediate Reaching>Baseline (t>8.5, corrected).

Recent evidence has shown that the human superior parieto-occipital cortex (SPOC) is involved in the arm transport component of reaching [39], [40]. We identified SPOC using a localizer contrast of immediate grasping and reaching vs. the intertrial baseline in 9 out of 11 participants of Group A (Figure 5 and Table 1E). The time course extracted from the experimental runs showed robust activation for the visual phase and the action phase (with higher activation for Go than Stop trials) but no delay period activation and no differences between grasping and reaching in any phase. These results are consistent with prior work showing no significant sustained activation in anterior SPOC during delayed reaching or saccades [41].

#### Dorsal Premotor Cortex

**Figure 6:**
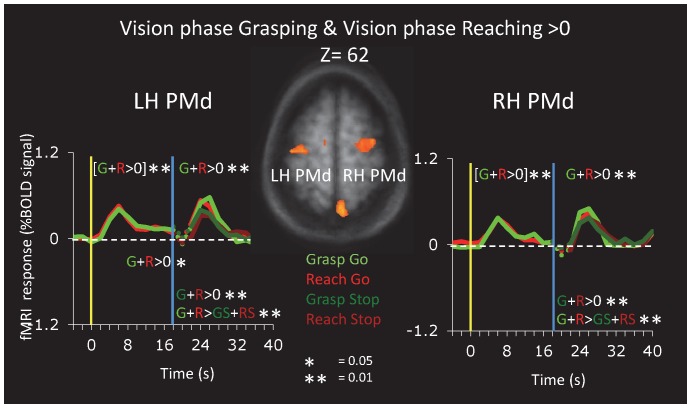
Activation in Ventral Premotor Cortex. Area PMd was identified in Group A (n = 9) using a contrast of Vision phase Grasping+Vision phase Reaching>Baseline (t>5, corrected).

Another area of interest is the dorsal premotor cortex, PMd, which is involved in both the transport and grip components of reach-to-grasp actions [40]. We identified PMd based on activation in the visual phase of the experimental trials in Group A (Figure 6 and Table 1G1F). PMd, like SPOC, showed activation for the visual and action phases of the trial; moreover, left PMd also showed significant sustained activation during the delay period.

### Exploratory Group Voxelwise Analyses

#### Relative contribution of vision and action

**Figure 7:**
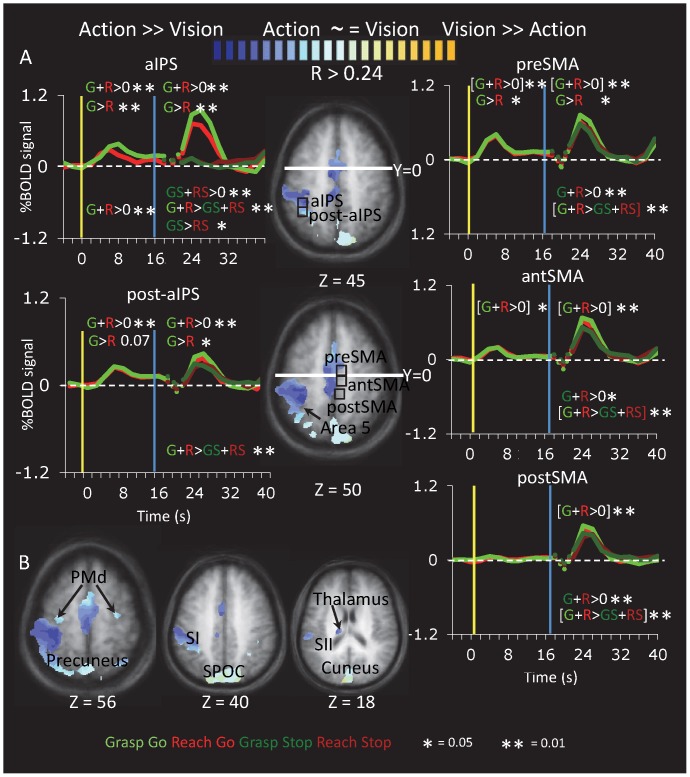
Relative Visual:Action Activation in Group-Averaged Data. Activation maps show the relative contribution between the visual and action predictors in a group voxelwise analysis (Group A, n = 11) shown on the group-average anatomical image for areas in which a gradient was observed (A) and other areas (B). For each voxel in which the visual and action predictors together accounted for a significant proportion of the variance (R >0.3), the figure shows the relative contribution index, computed the following contrast of beta weights: (+GG+RG-GV-RV)/(+GG+RG+GV+RV). Voxels with only responses to the action cues (grasping and reaching) appear dark blue; areas with only responses to the visual cues (both grasping and reaching) appear dark yellow; areas with comparable visual and motor responses appear in the intermediate range of the spectrum (light blue – light yellow). The time course for aIPS is the same as that in Figure 2A, repeated here to facilitate comparison with post-aIPS, the area immediately posterior to aIPS.

**Figure 8:**
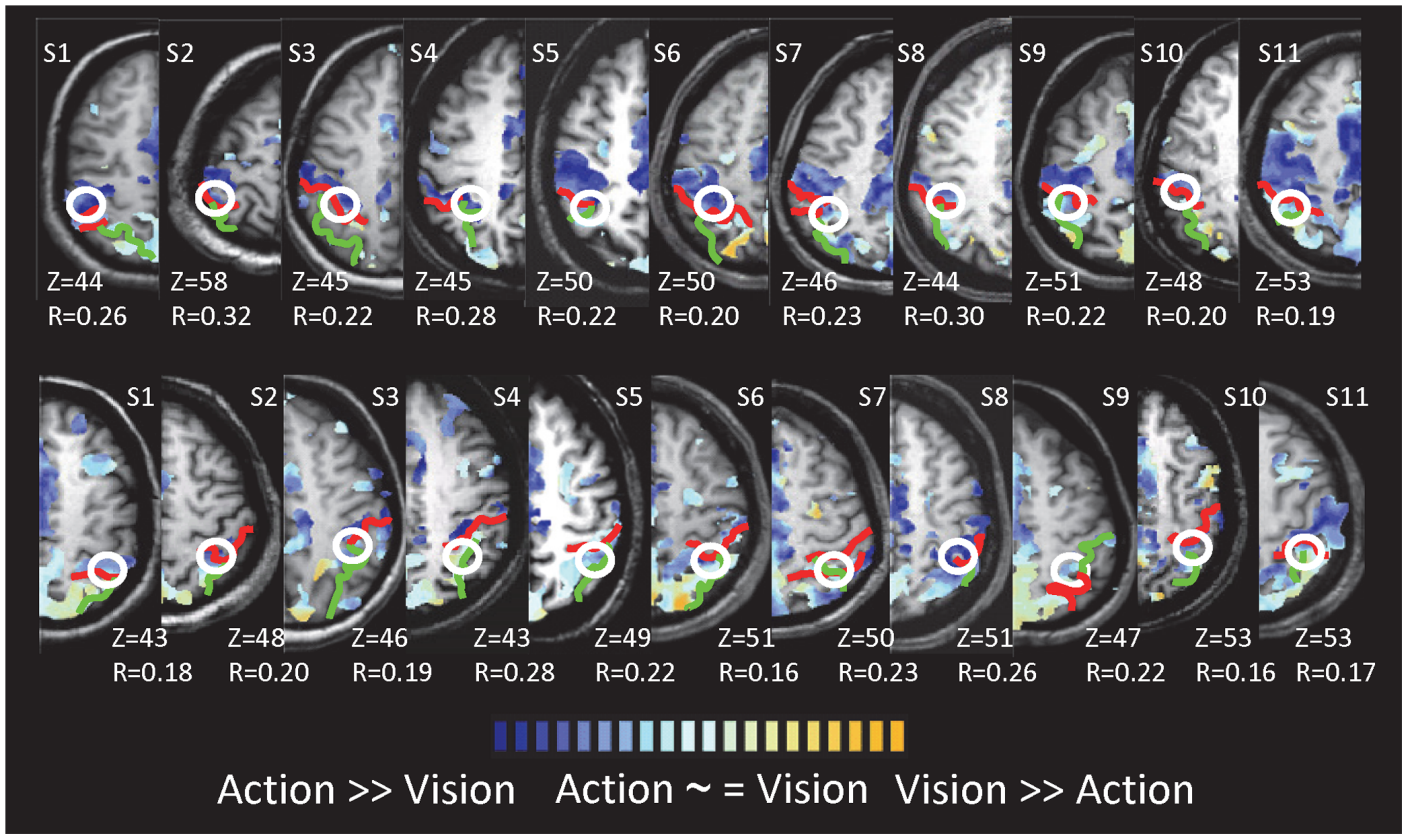
Relative Visual:Action Activation in Individual Subjects. The relative contribution map (as in Figure 7) is shown for each hemisphere in each participant. The postcentral and the intraparietal sulci are shown in red and green, respectively and the peak of area aIPS (based on the contrast of grasping>reaching from localizer scans) is circled in white.

The use of a delay paradigm allowed us to qualitatively evaluate the relative contribution of visual stimulation and motor execution (and/or memory recall) in reach to grasp actions. As shown in the activation map in Figure 7A, two zones showed a gradient between visual activation (for both grasping and reaching) and action (re-)activation (for Go trials for both grasping and reaching). First, there was a gradient within anterior parietal cortex. While both the anterior and posterior divisions of aIPS showed comparable visual responses, the anterior division showed a much more robust action-phase response. Of course, such a pattern could be observed simply because of intersubject variability of anatomical locations in stereotaxic space; that is, the more anterior voxels would be more likely to include primary motor cortex (M1) and primary somatosensory cortex (S1) while the more posterior voxels would be more likely to include other zones of the IPS. Thus we also examined the pattern in individual subjects, taking into consideration the location of aIPS as defined individually using the localizer data (Figure 8). Although there was considerable inter-individual variability, the majority of subjects showed an anterior-to-posterior gradient between action-only and both visual and action responses. Second, the group relative contribution map (Figure 7A) suggested a posterior-to-anterior gradient between action-only responses (posterior SMA, y <0) to both visual and action responses (pre-SMA, y <0). Other areas did not show any gradients (Figure 7B). Bilateral PMd, bilateral SPOC and precuneus, cuneus, and thalamus showed both visual and action responses. Primary (SI) and secondary (SII) somatosensory cortex, as well as area 5 showed predominantly action-related activation. Somewhat surprisingly, no areas showed predominantly visual activation, even within occipital cortex, suggesting that all areas with a visual response also showed (re)activation at the time of action execution. We generated Talairach coordinates for areas with responses to the Vision and Action (Go>Stop) conditions, as shown in Tables 1F and 1G.

#### Sustained delay period activation

**Figure 9:**
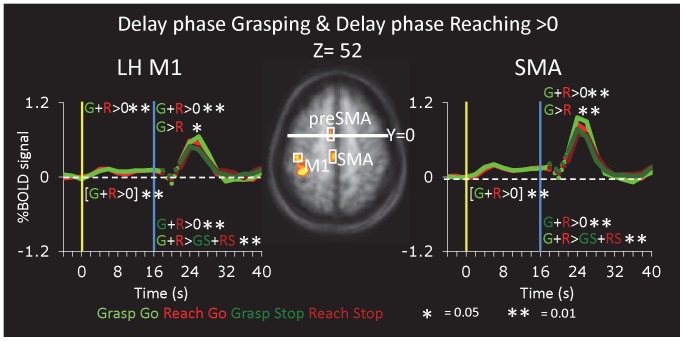
Activation During Delay Period. Areas SMA and M1 (second row) were identified in the subjects from Group A using the contrast Delay phase Grasping+Delay phase Reaching>Baseline (t>3.5, corrected).

In addition to enabling the separation of vision- and action-related activity, the long delay period allows us to identify areas with sustained delay period activation. When we contrasted delay period activation against the intertrial baseline [Grasp Delay+Reach Delay)>ITI], only three foci survived cluster threshold correction: pre-SMA, SMA and the central sulcus, presumably M1 (Figure 9 and Table 1H). Activation in left dorsolateral prefrontal cortex (DLPFC) was also observed but did not survive cluster threshold correction. Note that this is not entirely consistent with the conclusions one could draw from post hoc comparisons in areas defined by other criteria. These post hoc contrasts also indicated significant delay activation in bilateral aIPS (Figure 2A) and left PMd (Figure 6). The absence of significant delay period activation in these two areas with the voxelwise contrasts is likely due to the relatively more conservative nature of random effects contrasts (especially with our relatively small sample size, n = 11, and cluster-threshold correction) at the voxelwise level compared to the regional level.

## Discussion

A delayed action paradigm enabled us to examine the role of ventral- and dorsal-stream brain areas in visual processing, memory maintenance, and action execution requiring memory recall. First and most strikingly, we found that two putatively visual sensory areas – LOC and EVC – were not only activated during visual stimulus presentation as expected but were reactivated at the time of action *even when no visual stimulation was present*. Such results suggest that at the time of action execution, perceptual structures involved in object recognition and early visual processing are re-recruited to provide relevant information about object properties to guide the dorsal stream in performing the action. Moreover, stronger activation for grasping trials than reaching trials was observed in a subdivision of LOC (perhaps corresponding to LO_TV_) and MTG during execution and in EVC during both visual stimulation/encoding and execution/retrieval. Perhaps because grasping requires more detailed processing of visual features such as shape and size than does reaching, the perceptual system is more engaged during visual encoding and retrieval for grasping than reaching. Although our EVC activation focus falls within the cuneus near the calcarine sulcus, consistent with the expected location of dorsal V1 and/or V2 which represents the lower visual field, it is important to keep in mind that we did not obtain retinotopic mapping data, limiting our ability to conclusively specify the EVC area(s) involved. Second, our paradigm enabled us to dissociate visual responses from action execution responses within sensorimotor dorsal-stream areas. PMd, SPOC, and posterior aIPS all showed robust visual responses in addition to action execution responses, reinforcing their purported roles as *sensory-*motor (not simply motor) regions. In contrast, somatosensory areas (particularly S1and area 5) and the most anterior portion of aIPS showed responses primarily during action execution, when tactile and proprioceptive feedback would be available. Both posterior-to-anterior aIPS and anterior-to-posterior midline areas (pre-SMA to SMA) showed gradients between sensory-motor and predominantly motor activation. Third, sustained activation was observed throughout the long memory interval in a number of dorsal-stream areas, including aIPS, PMd, SMA, and even M1. Thus the human dorsal stream remains involved in maintaining action plans during a delay, consistent with human fMRI data showing motor planning activation while the object remains in view [42], [43] and with non-human primate neurophysiology [19]. Taken together, these findings enable us to propose a detailed, testable theoretical model of how ventral- and dorsal-stream areas contribute during delayed actions.

### Reactivation for Remembered Perceptions and Actions

Recent functional neuroimaging studies have provided growing support for an intriguing proposal with a long history including suggestions by Karl Wernicke [44] and William James [45]: memory retrieval invokes a re-excitation of the part of the brain that was initially excited by sensation. Early neuroimaging results revealed that during memory retrieval there is reactivation of modality-specific networks (e.g., visual, auditory or olfactory) activated during the initial stimulus [46]–[49]. Moreover, the reactivation of extrastriate regions during memory retrieval appears to reflect accurate reconstructions of the original visual categories and features [48], [50]–[52] and spatial locations [53].

Although most studies of memory reactivation have focused on the perceptual system, there have been some suggestions that similar reactivation processes are at play for memory-driven actions. For example, the recall of action phrases reactivates somatosensory and motor regions, particularly following performance of the action [47], [54]. In addition, remembered saccades yield retinotopically specific reactivation in EVC [55]. Here we extend this work to reaching and grasping, which are interesting because the introduction of a delay qualitatively affects performance in both neurologically intact research participants and neuropsychological patients [10].

### Elaboration of the Perception-action Model of Delayed Actions

In an influential explanation for the neuropsychological and kinematic differences between immediate and delayed actions, Goodale and colleagues [10] proposed that “the production of real-time actions to visible targets depends on pathways that are separate from those mediating memory-driven actions”. Our data not only reinforce those conclusions but enable us to outline a more detailed proposal of the specific human brain areas involved and their “choreography” during different phases of delayed actions.

We propose that at the time of the initial stimulus presentation, information enters the visual system predominantly through the primary visual pathway and activates areas in the ventral stream (including LOC) and the dorsal stream. As this visual response decays away, sustained responses are maintained in some areas of the dorsal stream (including SPOC, aIPS, PMd and the preSMA).

One framework that may be helpful in considering the types of sensorimotor processing occurring in later phases is the distinction between motor planning and motor programming (as an analogy, one may make a *plan* to drive to Toronto on the weekend but not *program* the specifics such as the route until the departure is imminent). Though speculative, we propose that during visual stimulation and the delay period, dorsal-stream areas may generate and maintain broad motor plans (e.g., to grasp a particular object with a precision grip). Once the cue to perform the action upon the remembered object is given, we propose that the specific details of the action may be programmed (e.g., specific positioning of the fingers on the object) and this programming requires that detailed visual information be reactivated via EVC and LOC (particularly LO_TV_). Interestingly, grasp-selective action re-activation extended into the MTG, an area activated by other fMRI tasks requiring crosstalk between the two streams: tool use [56] and pantomimed grasping [57]. Rerouting through the ventral stream could explain why delayed (vs. immediate) actions are more influenced by relational metrics (such as the relative object size) and scene-based coordinates [10].

Another useful distinction is between motor planning and motor control [58]. As a movement is executed, visual, somatosensory and proprioceptive feedback can be compared to a forward model of the expected movement (based on an efference copy of the movement) and used to correct errors [59]. Recent work from our lab has found surprisingly rich information about planned actions in occipitotemporal areas including LOC when sensory feedback is expected [60]. In this case, no visual feedback is provided during action execution; nevertheless, reactivation of visual areas may reflect recall of the visual details of the object such as size and comparison with feedback from proprioceptive and somatosensory systems.

We suggest that our data and model clarify the explanation of impairments in delayed grasping across a wide range of neuropsychological patients. For example, we suggest that the preservation of many dorsal stream areas within DF [6] enables her to maintain the correct task goals (e.g., precision grip) during the delay interval; however, due to her bilateral LOC lesions, she lacks the ability to re-recruit information about object size after a delay and thus fails to scale her grip. Similarly, the introduction of a delay impairs grip scaling in patients with EVC lesions [61], presumably because they are also unable to re-recruit all the information required for grip scaling. Interestingly, patients with hemispatial neglect also show impairments for delayed but not immediate actions [62]. Specifically, these patients, like DF, have poorer endpoint accuracy for reach-to-point movements when a delay is introduced. Though lesion sites for neglect patients are typically large and heterogeneous [e.g., 63, Figure 1], the lesion sites most strongly associated with reduced accuracy during delayed reaching were within occipito-temporal cortex, suggesting a loss of either the detailed information about location in the ventral stream or its ability to be relayed to the dorsal stream. Another interesting case is a patient with neglect who showed delay-specific grip scaling impairments [63] despite an intact ventral stream. One tentative explanation in this case may be that his lesions within parietal cortex may have disrupted the connections between the two streams such that even if size-specific information could be stored and recalled, it may not have been communicated to the dorsal stream at the time of action execution. Our interpretations are speculative but testable if patients who show delay-specific impairments can be tested for preserved functional activity (using fMRI) and connectivity (using resting state connectivity or diffusion tensor imaging).

The proposed model derived from this fMRI data has been reinforced by “virtual lesion” data using transcranial magnetic stimulation (TMS) to LOC and aIPS during immediate and delayed actions [64]. First, TMS to LOC disrupted early grip kinematics for delayed but not immediate grasping, supporting suggestions from the neuropsychological data that the reactivation observed in LOC is essential to successful grasping following a delay. That is, although the reactivation in areas like LOC (and EVC) may in part reflect processes such as mental imagery, the TMS data demonstrate that the activation is *causally* involved in successful performance and not merely an epiphenomenon of gratuitous mental imagery. Second, TMS to aIPS disrupted grip kinematics for both immediate and delayed grasping. This suggests that aIPS plays a role for both types of actions, although the literature is still somewhat equivocal about whether the dorsal stream is *more* involved for real-time than delayed actions. On one hand, several fMRI experiments have found comparable activation for immediate and delayed action execution [20], [21]. On the other hand, the motor system shows greater object-specific excitability when the part of the object to be grasped remains visually cued [65].

Our hope is also that a more detailed model will help to reconcile previously discrepant findings. For example, though we did not compare our delayed grasping activation to that for immediate grasping, our results are fully consistent with past fMRI studies showing strong recruitment of dorsal stream areas in both tasks [20], [21]. However, in addition to these past results, our data demonstrate re-recruitment of ventral-stream and EVC regions at the time of action. While these areas would no doubt also be found during immediate grasping, simply because the object is being visually processed, our data shows this reactivation occurs even without visual stimulation present and our model emphasizes the functional importance of this reactivation. Put another way, our results suggest that it is not that immediate actions rely on the dorsal stream while delayed actions rely on the ventral stream; rather both types of actions rely on the dorsal stream, but delayed actions also rely on the ventral stream.

One under-appreciated possibility in this literature is that types of information required by delayed actions and the areas involved may differ depending on task or stimuli. For example, the case for the recruitment of the ventral stream in delayed actions seems to be particularly strong for the role of size in grasping. Indeed, the strongest behavioral evidence for an abrupt transition to “ventral-stream mode” comes from size illusions in grasping [11], [24]. By contrast, accuracy in pointing tasks may show more graded effects of delay [16]. Our results here show which areas are implicated in size processing for delayed grasping but future studies could test other cases like location processing for delayed reaches or saccades. Grasping (grip) and reaching (transport) may rely to differing degrees on a dorsolateral substream (which includes aIPS) and dorsomedial substreams (which includes SPOC), respectively [40] and areas within these substreams may connect and communicate with ventral-stream and EVC regions in different ways.

### Remaining Issues

One remaining question is how brain areas in the two streams share information at the time of action. Some clues come from an event-related potential (ERP) experiment [66] that compared a condition in which reaching movements were programmed while the information was visually available (immediate) with a condition in which movements followed a very brief (<1-s) delay [24]. Compared to the condition with no delay, the condition that included a brief delay yielded a larger N170 ERP component – which is known to reflect a variety of ventral stream processes [66].

A related question is which areas of the frontoparietal dorsal stream network receive information from LOC and EVC. One candidate region is aIPS. The macaque brain contains direct connections between inferotemporal (IT) cortex and AIP [67], the putative homologues of LOC and aIPS, respectively. Moreover, these two macaque regions showed synchronized activity during anticipation of a visual stimulus in a simulated-3D shape discrimination task [68]. Taken together these anatomical and functional connections between the ventral (macaque IT or human LOC) and dorsal streams (macaque AIP or human aIPS) provide a plausible route by which visual object information could be relayed following a delay.

Another remaining question is what information is stored in various brain areas during the delay interval. Several dorsal-stream areas showed significant delay period activation that was comparable for grasping and reaching. Specifically sustained delay-period activation was observed in SMA, pre-SMA, and M1 (in voxelwise analyses) along with aIPS and PMd (in the ROI analysis). Although the delay-period activation in aIPS is consistent with its putative homology with macaque AIP [19], it begs the question of what the elevated delay-period activity in both species is coding. A related puzzle is why this representation (at least in patients like DF) does not appear to be sufficient to plan a fully formed grasp (taking into account grip size for example). Certainly a number of studies have demonstrated selectivity to disparity-defined 3D-shape in macaque AIP [69]–[72] and F5a [33], as areas just posterior to human aIPS (e.g., dorsal IPS anterior, DIPSA, and dorsal IPS medial, DIPSM [73]). However, these studies have only looked at the neural responses and selectivity during visual stimulation and not during a delay period. In macaque AIP, the testing of a subset of neurons (6 of 18) retained their shape-selectivity over a delay interval (e.g., a neuron that fired most strongly to a horizontal plate compared to other objects also showed the strongest firing for a horizontal plate during the delay period) [19]. Macaque AIP also retains some orientation preferences during a delay period [74]. We speculate that during the delay interval, dorsal-stream areas may be coding the broad motor-related parameters of the grasp related to the grip (e.g., grip type) but without coding for some finer details about the object (e.g., size), thus requiring input from other regions LOC and EVC at the time of action programming and execution.

In considering neural processing during the delay period, it is important to note that neural coding may not necessarily be manifested as increased BOLD activation (relative to the intertrial interval). For example, pattern classifiers applied to fMRI activation during memory delays [75]–[77] have found that rich information can be stored even in areas that show negligible delay activity [78], [79]. Conversely, even when delay activity is present, it may not enable decoding of the remembered stimulus [80].

Although our data did not include enough trials to apply pattern classification, information about the upcoming trial type may be decodable during the delay period as it is during a planning period in which the stimulus remains visible [43]. It may also be possible to disrupt an upcoming movement with TMS to areas like contralateral V1 and LOC during the delay period, just as TMS to V1 and dorsal-stream areas during a delay period disrupts performance on other tasks [18], [81], [82].

### Conclusions

In sum, these data have led to a detailed, testable model of how delayed actions may rely on brain areas within the ventral and dorsal visual streams. Indeed, two follow-up experiments using TMS [64] and ERPs [66] have already found data consistent with our predictions. Ongoing experiments will investigate neural coding during the delay period, the causal role of additional regions in kinematics of immediate and delayed grasping, and functional/effective connectivity between brain areas. Undoubtedly, further experiments will lead to revisions and refinements to the model proposed here. Nevertheless, the data shown here provide a more detailed account than past theories regarding how specific brain regions within the two visual streams contribute to the phases of delayed actions.
